# Genome Sequence of *Enterobacter cloacae* Strain SENG-6, a Bacterium Producing Histo-Blood Group Antigen-Like Substances That Can Bind with Human Noroviruses

**DOI:** 10.1128/genomeA.00893-16

**Published:** 2016-08-25

**Authors:** Satoshi Ishii, Mohan Amarasiri, Satoshi Hashiba, Peiyi Yang, Satoshi Okabe, Daisuke Sano

**Affiliations:** a Department of Soil, Water and Climate and Biotechnology Institute, University of Minnesota, St. Paul, Minnesota, USA; b Division of Environmental Engineering, Hokkaido University, Sapporo, Hokkaido, Japan

## Abstract

*Enterobacter* sp. strain SENG-6, isolated from healthy human feces, produces histo-blood group antigen (HBGA)-like substances that can bind with human noroviruses. Based on the genome sequence analysis, strain SENG-6 belongs to the species *Enterobacter cloacae*. The genome sequence of this strain should help identify genes associated with the production of HBGA-like substances.

## GENOME ANNOUNCEMENT

Human noroviruses are the leading cause of nonbacterial gastroenteritis (1) and utilize histo-blood group antigens (HBGAs) as binding receptors to infect human cells (2). Because some Gram-negative bacteria were reported to have blood group activity (3), we screened enteric bacteria that carry HBGA-like substances from human feces by using anti-HBGA antibodies to see if these bacteria can capture human noroviruses (4). One such strain, *Enterobacter* sp. strain SENG-6, showed strong binding activity to norovirus-like particles (4). The HBGA-like substances are located in the extracellular polymeric substances (EPSs) secreted from strain SENG-6; however, the genetic mechanism associated with the production of HBGA-like substances is largely unknown. Genome sequencing of strain SENG-6 was conducted to identify genes associated with the production of HBGA-like substances.

*Enterobacter* sp. strain SENG-6 was grown in Luria-Bertani medium overnight at 37°C, and genomic DNA was extracted using the DNeasy blood and tissue kit (Qiagen). Sequencing libraries were prepared using the TruSeq DNA sample prep kit (Illumina) according to the manufacturer’s instructions. The genome was analyzed using the Illumina HiSeq 2000 with a 101-bp paired-end library. The resulting high-quality sequences (44,316,264 reads) were assembled using Velvet version 1/2/08 (5) to a total length of 5,030,416 bp, providing approximately 889-fold genome coverage. Gene prediction and annotation were performed by the NCBI Prokaryotic Genome Annotation Pipeline. Average nucleotide identity (ANI) was calculated using JSpecies (6).

Annotation of the *Enterobacter* sp. strain SENG-6 genome identified 4,824 genes, 4,650 protein-coding sequences, 14 rRNAs (seven copies), 73 tRNAs, five noncoding RNAs (ncRNAs), and 82 pseudogenes. The average GC content was 53.2%, which is comparable to other *Enterobacter* spp. (7). The ANI value between the genomes of strains SENG-6 and *Enterobacter cloacae* strain ATCC 13047^T^ (8) was 98.5%, which is greater than the cutoff value for species discrimination (95 to 96%) (6, 9). This result, together with the highly similar 16S rRNA gene sequences between strains SENG-6 and ATCC 13047^T^ (99.9%), suggests that strain SENG-6 belongs to the species *Enterobacter cloacae*.

The genome of strain SENG-6 contains genes encoding various enzymes for amino-sugar and glycan biosynthesis, which is likely involved in the production of HBGA-like substances. *Enterobacter cloacae* strain SENG-6 expresses A, B, and H antigens in EPSs (4), while *Enterobacter cloacae* (ATCC PTA-3882) has been found to carry an H antigen and promote human norovirus infection of B cells *in vitro* (10, 11). Another strain of *Enterobacter cloacae* (LMG2783) does not express A, B, and H antigens but has Le^a^ and Le^b^ antigens and contributes to the thermal stability of human norovirus particles (12). These suggest that HBGA expression profiles may vary among strains of this species. Comparative genomics of these strains may allow us to identify the genes associated with the production of the HBGA-like substances that can bind with human noroviruses.

### Accession number(s).

This whole-genome shotgun project has been deposited at DDBJ/ENA/GenBank under the accession number LOMM00000000. The version described in this paper is the first version, LOMM01000000.
